# Developing a Fecal DNA Method With Blocking Primers to Determine Red Knots' (*Calidris canutus*) Diets Noninvasively

**DOI:** 10.1002/ece3.74283

**Published:** 2026-09-15

**Authors:** Christiana K. Teye, Erin K. Grey, Alan Kneidel, Stephen Brown, Liana DiNunzio

**Affiliations:** ^1^ University of Maine Orono Maine USA; ^2^ Manomet Conservation Sciences Plymouth Massachusetts USA

**Keywords:** blocking primer, dietary assessment, eDNA, horseshoe crab, host DNA, PCR, prey DNA, Red Knot

## Abstract

Understanding the diets of migratory shorebirds such as the Red Knot *(Calidris canutus)* is essential for evaluating habitat quality, informing conservation policies, and monitoring ecosystem health. Fecal DNA can be complementary to traditional approaches to understanding shorebirds' diets, as it offers a noninvasive, efficient, and high‐resolution alternative for dietary analyses. However, one significant challenge with fecal DNA is the presence of host DNA, which can dominate polymerase chain reaction (PCR) amplification, hindering the detection of ingested prey DNA. In this study, we designed a blocking primer that reduced DNA amplification in Red Knots ( *Calidris canutus* ) by 68.3% in the absence of the blocking primer to 34.6% when the primer was used (paired *t* = 4.11, df = 10, *p* = 0.002; Wilcoxon signed‐rank *p* = 0.003). The method was validated on field samples (*n* = 52) from Cape Cod, MA, and multiple prey taxa were identified, including horseshoe crab eggs, a major prey item in this area. The success of this study confirms the utility of blocking primers in dietary assessment involving host‐dominated eDNA samples.

## Graphical abstract

The blocking primer suppressed Red Knot DNA while improving detection of other prey taxa. We detected horseshoe crab in the Fall fecal samples, suggesting their use goes beyond the spring migration period of the Red Knots.

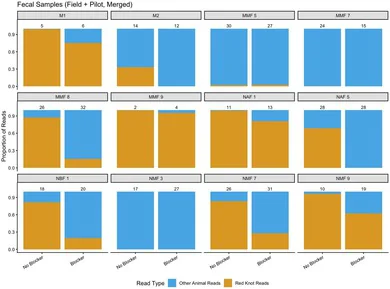

## 1 Introduction

Migratory shorebirds depend on high‐quality food resources at staging sites to refill fat reserves that are necessary for their long‐distance migrations (Baker et al. 2004). Their diet composition directly influences energy intake, fat storage, migration success, and overall survival. In contrast, insufficient prey availability can delay their departure from staging areas, reduce their endurance during migration, and reduce their reproductive success (Piersma 2003). Thus, understanding the diets of migratory shorebirds is essential for evaluating habitat quality, informing conservation policies, and monitoring ecosystem health. This knowledge helps conservationists assess whether key staging sites provide sufficient prey for migration (Van Gils et al. 2003).

Red Knots, *Calidris canutus* , are medium‐sized shorebirds known for their long‐distance migrations. Of the six subspecies of Red Knot, the *rufa* subspecies (*Calidris canutus rufa*, hereafter referred to as *rufa* Red Knot) migrates the longest distance, capable of trips totaling 30,000 km from their breeding grounds in the high Arctic to their wintering grounds in South America (Niles et al. 2010). During their migrations, Red Knots use staging sites (Piersma et al. 2016) to rest and rebuild fat reserves needed to fuel migration (MacDonald et al. 2021). Horseshoe crab (*Limulus polyphemus*) eggs are an example of an essential food resource for *rufa* Red Knots during migration stopovers along the North American Atlantic coast, helping them reach the body mass needed for successful migration and survival (Duijns et al. 2017). Habitat present at staging areas includes ocean‐ and bay‐front areas, barrier islands, mainland beaches, shorelines, and tidal flats (U.S. Fish and Wildlife Service [USFWS] 2021). Many staging areas face pressures from development and sea‐level rise, which can cause significant declines in shorebird populations (Iwamura et al. 2013; Chan et al. 2019). For example, a critical northbound staging area for the *rufa* Red Knot, which is listed as threatened under the U.S. Endangered Species Act (Edenfield and Edenfield 2024), is Delaware Bay, DE, USA.

For this study, we developed and used an eDNA blocking primer to assess the *rufa* Red Knot diet at the Monomoy‐Pleasant Bay system on Cape Cod, Massachusetts, a staging area used during southbound (fall) migration. This area provides critical resources for an estimated 1500–2000 knots annually, down from an estimated 5000 knots a decade ago (Harrington, Hill, and Nikula 2010; Harrington, Koch, et al. 2010). It is unclear whether these declines are due to reduced suitability of the staging area, overall population decline, or the adoption of new staging areas outside of Massachusetts. Like many coastal staging areas, this region is experiencing pressure from aquaculture, fisheries, recreational disturbance, and climate change (Smith 2015). Dietary insights are critical under these additional pressures, as they may alter prey abundance and distribution, thereby affecting the resilience of shorebird populations (Iwamura et al. 2013). Accurately identifying shorebird prey can help researchers understand why these birds are declining and how this decline affects ecosystem functioning (Hamilton et al. 2006). While there have been observational studies published on foraging patterns of Red Knots on Cape Cod (Harrington, Hill, and Nikula 2010), there have been no quantitative prey studies done in relation to shorebird distribution in this area.

Shorebird prey items may be found on the surface of or buried deep in their foraging areas, and are swallowed whole, crushed in the gizzard, and ejected as feces (Piersma et al. 1993). Traditional methods for identifying prey items foraged by shorebirds vary in data quality and in their impact on shorebirds. One traditional method is direct observation, in which observers watch birds forage and record their observations. This noninvasive method can only determine the general feeding behavior and is limited to visible prey items. Small, easily ingested prey can be overlooked, leading to an incomplete dietary assessment (Goss‐Custard et al. 2006). Another noninvasive method involves examining shorebird excreta to identify undigested prey items (Zwarts and Wanink 1993). This approach underrepresents soft‐bodied prey and requires a high taxonomic resolution for accurate dietary assessment (Barrett et al. 2007). Other methods, such as gut sampling, are more invasive, require capturing birds for stomach flushing, and pose a risk of injury or death (Tsipoura and Burger 1999). Environmental DNA (eDNA) metabarcoding has emerged as an alternative that can overcome some of the shortcomings of traditional techniques. It provides a sensitive, noninvasive approach by detecting traces of DNA shed by a target species in its environment. In this approach, the genetic material can be obtained with or without the source organism present. Available prey can also be identified by collecting DNA from water samples, sediments, feces, air, soil, and other environmental substrates (Thomsen and Willerslev 2015). Although “eDNA” is sometimes reserved for DNA collected from environmental substrates without the organism being present, in this study we use the term “fecal DNA metabarcoding” to refer specifically to prey DNA recovered from Red Knot fecal samples, consistent with conventional usage in the field.

Although eDNA metabarcoding has proven effective in a variety of studies, it still presents challenges, particularly for dietary analyses using fecal samples. One significant challenge is the presence of host DNA, which can dominate polymerase chain reaction (PCR) amplification, hindering amplification of ingested prey items (Deagle et al. 2007). This is a significant issue for shorebirds and most animals, as their fecal samples contain a substantial portion of their DNA (Pompanon et al. 2012). Using primers that amplify host DNA often biases amplification away from low‐concentration prey DNA, leading to uninformative sequence analyses, as reference sequencing libraries predominantly yield host reads (Pompanon et al. 2012). To address this challenge, blocking primers can be designed to bind host DNA without amplifying it, thereby inhibiting host DNA amplification and increasing prey DNA amplification (Vestheim and Jarman 2008).

In addition to primer design, efficient extraction of high‐quality DNA is an essential step in environmental DNA studies, as it can influence subsequent processes, including amplification success, sequencing, and, to some extent, taxonomic resolution (Deiner et al. 2017). At the same time, eDNA samples, especially those obtained from fecal and sediment sources, typically contain low concentrations of target DNA and substantial amounts of PCR inhibitors, such as bile salts, which often reduce detection sensitivity (Goldberg et al. 2016). Most studies have confirmed that the choice of extraction method can notably affect DNA yield, purity, and taxon representation in metabarcoding datasets (Tsuji et al. 2019). Selecting and optimizing extraction kits and protocols to suit the sample type are vital for maximizing prey detection and, most importantly, reducing bias and error (Ruan et al. 2022). To do this, commercially available extraction kits are tested and compared using fecal and sediment samples to identify which kit yields the highest‐quality DNA for dietary assessments (Ruan et al. 2022).

In this study, we aimed to (1) design and develop blocking primers to reduce amplification of Red Knot DNA and increase prey DNA amplification, (2) test DNA extraction kits for fecal and sediment samples, and (3) validate the blocking primer assay on fecal and sediment samples of the *rufa* Red Knot from various locations within the Monomoy‐Pleasant Bay system of Cape Cod, Massachusetts. We predict that blocking primers will increase the relative abundance of potential prey reads and that the field survey will yield realistic prey species for this migratory shorebird.

## 2 Methods

### 2.1 DNA Extraction Kit Test

Three different extraction kits were used to extract DNA from fecal (*n* = 9) and sediment samples (*n* = 9) collected from Monomoy National Wildlife Refuge, Massachusetts, on September 21st, 2021, following the manufacturer's instructions. To test for differences in DNA yield among the kits, each sample was subsampled and extracted on three kits: the Qiagen PowerSoil Pro Kit (Qiagen), the Zymo Midi Kit (Zymo), and the Zymo Mini Kit (Zymo). For the Qiagen PowerSoil and Zymo Mini Kit, 0.5 g of each fecal sample or 0.25 g of each sediment sample was weighed and dried for approximately 2 min. For the Zymo Midi Kit, 5 g of fecal or sediment sample was weighed. DNA concentrations were estimated using a Qubit 4.0 fluorometer (ThermoFisher) with the 1× dsDNA High Sensitivity kit (ThermoFisher) according to the manufacturer's instructions.

To test for differences in community composition between the Qiagen Powersoil and Zymo Midi kits, which yielded the highest fecal and soil DNA concentrations, respectively, we metabarcoded six samples extracted with both kits using the general metazoan COI primers mlCOIintF and jgHCO2198 (Leray et al. 2013). For metabarcoding in this experiment, we used a 2‐step approach, with the first PCR including the primers and the Nextera adapter. Each 25 μL PCR reaction consisted of 1× GoTaq Flexi Buffer (Promega), 0.625 units GoTaq Flexi DNA polymerase (Promega), 1 mM MgCl_2_ (Promega), 0.2 mM dNTPs (New England Biolabs), 0.12 mg/mL BSA (New England Biolabs), and 1 μL template DNA and underwent the following thermocycling program: initial denaturation at 95°C for 3 m, 16 cycles of 95°C for 15 s, 62°C for 30 s minus 1°C each cycle, 72°C for 60 s, 25 cycles of 95°C for 15 s, 46°C 30 s, 72°C 60 s, and final extension at 72C°C 3 m. For quality control, we included a no‐template blank control and a mock community positive control consisting of DNA tissue extracts from species not known in New England waters (Neon Tetra fish, *Paracheirodon innesi* , Gulf shrimp, *Panaeus vannamei*, Argentine shortfin squid, *Illex argentinus* , Queen scallop, *Aequipecten opercularis* , and Bobcat, *Lynx rufus* ).

Amplicons were sent to the University of Rhode Island's Molecular Informatics Core (MIC) for library preparation and sequencing with Illumina MiSeq Reagent Kit v3 2 × 300 bp (Illumina). MIC library preparation included size‐selection cleanup with Ampure XP Beads (Beckman Colter), indexing PCR with Phusion HF premix (ThermoFisher) and Nextera Indices (Illumina), pre‐pooling DNA quantification with a Qubit fluorometer (ThermoFisher), pooling of samples in equimolar concentrations, library quality check with a BioAnalyzer DNA100 chip (Agilent) and KAPA quantification kit (Roche), and a PhiX spike (Illumina) to balance fluorescent signals.

### 2.2 Blocker Primer Development

Two commonly used general animal primers, “Leray” COI primers mlCOIintF/jgHCO2198I (Leray et al. 2013) and “TarEuk” 18S V4 primers TAReuk454FOR1/TAReukREV3 (Stoeck et al. 2010), were both found to amplify Red Knot DNA from tissue and fecal samples collected from Massachusetts. For blocking primer development, we selected the Leray COI primer set because it generally exhibits greater species specificity than the TarEuk 18S primer set in metazoans (Giebner et al. 2020).

To design this blocking primer, we first aligned the forward and reverse primers of the universal Leray COI primer set (forward primer mICOIintF: 5′‐GGWACWGGWTGAACWGTWTAYCCYCC‐3′, reverse primer jgHCO2198: 5′‐TANACYTCNGGRTGNCCRAARAAYCA‐3′) onto the Red Knot COI Accession # MK262432 in Benchling. Several potential blocking primers that overlapped ~10 bp with the forward primer of the Leray primer sets and extended ~10–15 bp into the Red Knot COI sequence were identified visually in the alignment. These blocking primer candidates were then ordered from IDT with various spacer CPG or inosine repeat configurations and tested for their ability to reduce Red Knot amplification while maintaining amplification of other invertebrate species. Test PCRs used Red Knot tissue DNA, *Mytilus* tissue DNA samples, a Mock community [having tissue DNA from these species: Argentinian Squid ( *Illex argentinus* ), Whiteleg Shrimp ( *Litopenaeus vannamei* ), Neon tetra ( *Paracheirodon innesio* ), Queen scallop ( *Aequipecten opercularis* ), Gulf Shrimp ( *Farfantepenaeus aztecus* ), Bobcat ( *Lynx rufus* ), Platy (*Xiphophorous* sp)], and study site sediment eDNA samples as templates, with reaction mixes (each sample consisted of 0.7 μL of PCR water, 10 μL of repliQa HiFi ToughMix, 8 μL of 5 μM of the candidate blocking primer, 0.8 μL of 5 μM of mlCOIintF/jgHCO2198I primers, 0.5 μL of the DNA template) and thermocycling parameters (98°C for 1 min, 98°C for 10 s, 62°C (−1°C) for 5 s, and 68°C for 2 s, and this ran for 16 times, 98°C for 10 s, 46°C for 5 s, and 68°C for 2 s, and this ran for 25 times).

Among the candidate blocking primers tested, the F2S (5′‐CAGTATACCCCCCACTCGCTGG/3SpC3/‐3′) was selected based on its ability to strongly reduce Red Knot DNA amplification while not greatly affecting amplification of the invertebrate test templates (Mytilus tissue DNA, mock community, and sediment eDNA) as assessed through visual inspection of electrophoresis gel bands.

### 2.3 Field Validation of Metabarcoding Assay

#### 2.3.1 Sample Collection

**FIGURE 1:**
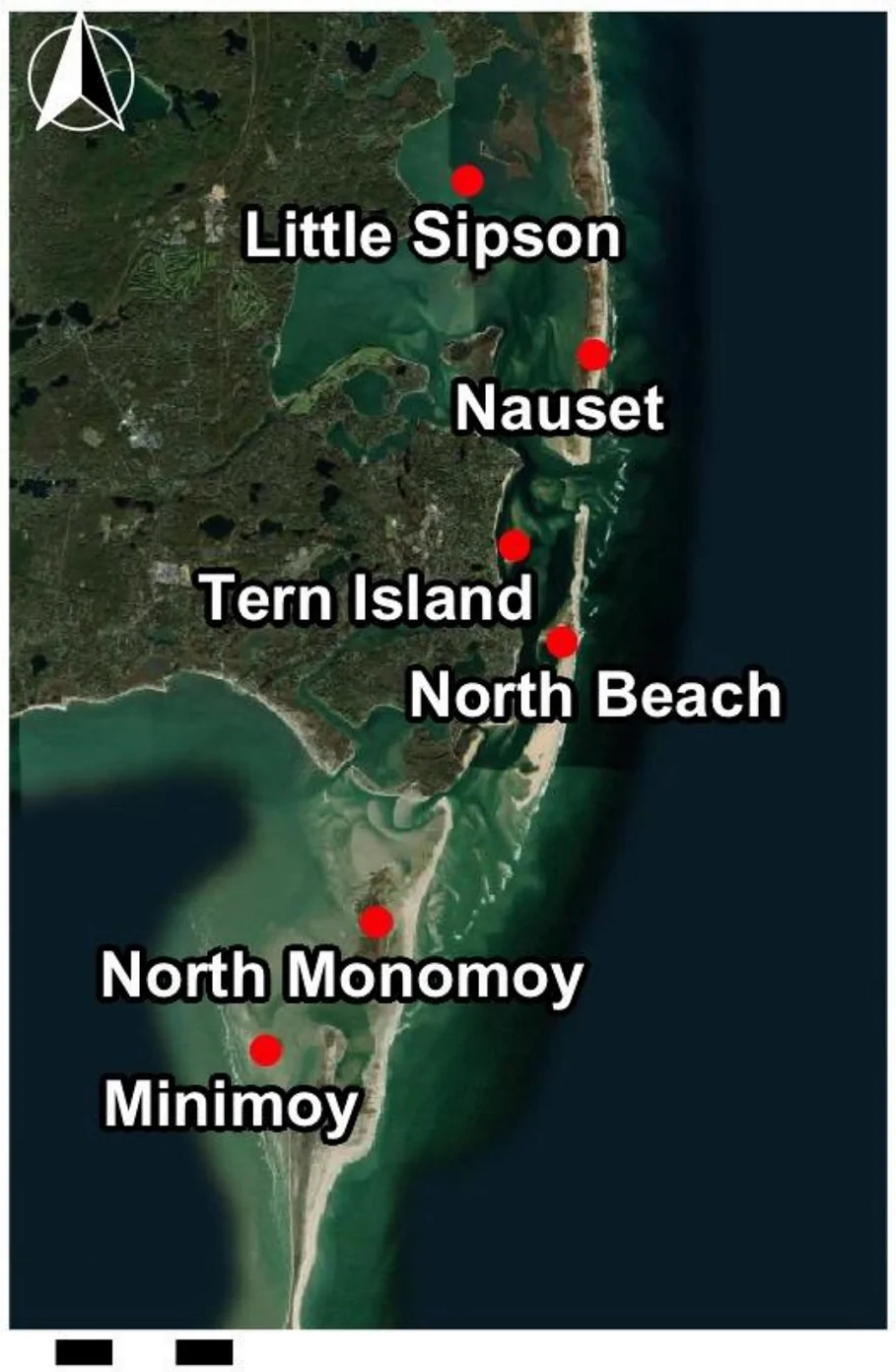
A map showing the sampling sites listed in Table 1. This plot was generated in R version 4.3.2, using the sf (Pebesma 2018) and rnaturalearth (South 2017) packages.

For field validation of the blocking primer metabarcoding assay, sediment and fecal samples were collected from Red Knot foraging grounds within the Monomoy‐Pleasant Bay system in Cape Cod, Massachusetts (Figure 1). We collected samples at low tide when the mudflat foraging areas were exposed. These samples were collected approximately once per month from July to November 2022. We randomly selected 19 sediment sampling points distributed across six sites (Table 1). Sediment samples were collected from the sampling points using Terra Core Soil Samplers (5–10 g samples) and stored in 5.0 mL vials. If Red Knots were present at the sampling sites, we also collected fecal samples. This involved observing foraging Red Knots and collecting fecal samples from the mudflats using 1.5 mL vials. Care was taken not to collect samples from other shorebird species by only collecting from pure Red Knot flocks or by watching individual knots closely and collecting samples immediately after defecation. The collected samples were stored in ethanol and shipped to the University of Maine, Orono, USA, for further analysis.

**TABLE 1:** Sites at which sediment and fecal samples were collected for dietary assessment of the Red Knot ( *Calidris canutus* ) using eDNA, Cape Cod, Massachusetts, USA, in 2022.

Sites	Fecal sample count	Sediment sample count
Nauset	6	13
North Beach	2	6
North Monomoy	14	32
Minimoy	10	13
Tern Island	0	6
Little Sipson	0	6

With these field samples, the metabarcoding assay was assessed in two ways. First, we tested the F2S blocking primer's effectiveness in reducing host DNA amplification and increasing prey DNA detection by amplifying a subset of fecal (*n* = 12) and sediment (*n* = 12) samples, with and without the blocking primer. Secondly, we assessed the Red Knot diet on Cape Cod by amplifying all fecal and sediment samples using the Leray and F2S primers. Both the blocker test and the full‐field survey were metabarcoded as described below.

#### 2.3.2 Metabarcoding

For the field assessment, the COI regions of the DNA extracts were amplified using a Bio‐Rad C1000 Touch thermocycler with the Leray primer set plus Red Knot blocker F2S. Each PCR reaction consisted of 10 μL of RepliQa HiFi ToughMix 2×, 4.8 μL of 5 uM F2S blocking primer, 0.8 μL of 5 μM Leray primer, 1 μL of the DNA extract, and 3.4 μL of PCR water, for a total of 20 μL per reaction. The thermocycling program was 98°C for 1 min, 98°C for 10 s, 62°C (−1°C) for 5 s, and 68°C for 2 s, and this ran for 16 times, 98°C for 10 s, 46°C for 5 s, and 68°C for 2 s, and this ran for 25 times. Positive (Red Knot tissue DNA, Mock community) and negative control samples (water) were included to assess contamination.

Amplification was checked via gel electrophoresis on a 2% agarose gel stained with GelRed. Three microliters of the amplified DNA were mixed with 2 μL of loading dye and then loaded into the gel wells. The samples were run at 135 V for 20 min in 1× TE buffer. The gels were visualized using the Azure300 gel imager. The amplicons were stored at −20°C.

Before sequencing, the sediment amplicons were pooled by collection date and sampling site, with *n* = 3 from Little Sipson, *n* = 4 from Minimoy, *n* = 3 from Nauset, *n* = 3 from North Beach, *n* = 4 from North Monomoy, and *n* = 3 from Tern Island. Pooled sediment and all individual fecal samples were sent to the University of Rhode Island's MIC for library preparation and sequencing as described above in Section 2.2.

### 2.4 Bioinformatics

The same bioinformatics pipeline was used for the blocker test, the extraction kit test, and the field‐test metabarcoding runs. Demultiplexed FastQ files were downloaded from Illumina BaseSpace, and raw read quality was visually assessed using FastQC (Andrews 2010). Adapter and primer sequences were trimmed using *cutadapt 5.2* (Martin 2011), and reads were retained if both primer sequences were successfully trimmed. DADA2's filterAndTrim was used with truncLen = c(200,150), maxEE = c(2,5), default truncQ = 2, to trim the reads, which were filtered for quality, dereplicated, denoised to Amplicon Sequence Variants (ASVs), merged, and the chimera removal step was performed using the default consensus method in removeBimeraDenovo (Callahan et al. 2016).

Taxonomy was assigned to each ASV to the lowest possible level using two approaches: the first approach used the *dada2 assignTaxonomy* function and a custom reference library created for the Gulf of Maine (Davis et al. 2024), and the second approach was a local BLAST (Camacho et al. 2009) of ASV sequences to the MIDORI2 UNIQ NUC GB260 CO1 BLAST reference library (Leray et al. 2022). Final taxonomic assignment for each ASV was determined based on a series of decisions that considered concordance between the two methods and match quality. A taxonomic rank was assigned to an ASV when both the BLAST and DADA2 methods agreed. If there was disagreement at the species level and the DADA2 species bootstrap value was > 0.80, the DADA2 species assignment was used. If the DADA2 species bootstrap was < 0.80 but the BLAST assignment was > 0.98 identity and > 200 bp match length, the BLAST species assignment was used. The ASVs with species assignment sequence identities < 0.98 but > 0.90 were assigned to the genus. For the contamination step, we used the maximum read count observed in the negative control as the subtraction threshold, which flagged 173 ASVs as background, removing 841,435 reads (25.8% of 3,256,923 total) across 1218 sample × ASV cells. The resulting ASVs with counts < 0 were removed. The entire bioinformatic workflow for each run, including parameter values and a summary of reads at each step for each sample, is available at https://github.com/egreyavis/REKN_blocker_dvlpmt.git.

### 2.5 Quantitative Analyses

To test differences in blocking primer effects, the extracts and the mock sample were amplified with and without the blocking primer, and the resulting amplicons were sequenced. For each sample, the total number of reads assigned to the host was summed and divided by the total number of reads in the sample to calculate the proportion of host DNA. The proportion of prey reads was subtracted from the host read count, and the difference was used to assess the percentage reduction and the blocking primer's efficiency in reducing host DNA amplification.

To assess differences among the extraction kits, DNA concentrations of the extracts were measured using a Qubit 4.0 fluorometer (Invitrogen) with the 1× dsDNA High Sensitivity kit. Measurements were expressed as ng/μL, and an ANOVA was conducted to assess the effects of the kits on DNA concentrations. To compare metabarcoding results, qualitative visual analyses of ASV accumulation by read count were used to assess sequencing depth. A *t*‐test was used to assess the effect of the extraction kit on observed ASV richness, Chao1‐predicted ASV richness, and Shannon diversity. Finally, an Analysis of Similarities (ANOSIM) test was conducted to assess the effects of kits on community composition using both Jaccard and Bray–Curtis dissimilarity metrics. Accumulation curves, richness and diversity estimates, and ANOSIM analyses were conducted using the *vegan* package (Oksanen et al. 2025).

For the field test of the F2S blocking primer, we used paired *t*‐tests to assess the significance of differences in the proportion of Red Knot reads, ASV richness, and Shannon diversity between unblocked and blocked amplicons, and then PERMANOVA to test for differences in community composition between these treatments.

To determine whether the blocking primer's effect on prey detection was the same across all species or varied by species, all prey species detected in the fecal field samples were ranked by total read abundance. The five most abundant species were chosen for the fold‐change analysis. For each of these species, the relative abundance per sample was calculated by dividing the number of reads for the species by the total number of animal reads in that sample. Samples were paired according to the time and place they were collected (i.e., those collected with the blocker and those collected without it), and the fold‐change was computed for each pair as the ratio of the relative abundance with the blocker to the relative abundance without the blocker. For each species, we recorded the number of paired samples in which it was detected, the mean relative abundance with and without the blocker, the mean fold‐change across the pairs in which the species was present in at least one of the conditions, and the number of pairs in which the species went from being present (in the samples without the blocker) to completely absent (in the samples with the blocker).

For the full‐field survey, the phyloseq package (McMurdie and Holmes 2013) was used to filter ASVs to include only animal phyla. Only the eight most abundant animal classes were used for further analysis. To compare communities across field samples, we used the transform_sample_count() function in the phyloseq package in RStudio to normalize read count data to relative abundances by dividing each sample count by the total across all samples. To visualize community patterns across sites and sampling dates, the relative abundances of the top 30 taxa were shown in stacked bar plots. These relative abundance bar plots were made for fecal and sediment samples by site over time (Figures 5 and 6). For the fecal samples, we calculated the host DNA ratio in each sample by dividing the number of reads assigned to the Red Knot by the total number of animal reads. We then compared the paired means between the Blocker and No Blocker conditions, both across all samples and only among those where host DNA was detected.

We calculated prey ASV richness (specnumber) and Shannon diversity (diversity, index = “shannon”) from the prey community matrix, excluding host DNA and Aves, for both fecal and sediment samples. To compare richness and diversity between Blocker and No Blocker conditions for each sample type, we used paired *t*‐tests and Wilcoxon signed‐rank tests. We assessed community composition using Bray‐Curtis dissimilarities and applied PERMANOVA (adonis2, 999 permutations), specifying sample pairs as strata to account for the paired design. Beta‐dispersion (betadisper, permutest) was used to test if variation in composition differed between Blocker and No Blocker samples. For fecal samples, we also compared paired richness after rarefying to the same sequencing depth (rarefy), excluding pairs where prey read depth was below the minimum threshold in one condition.

To see if the blocking primer affected prey detection equally across all taxa or varied by taxon, we ranked all prey species detected in the fecal samples by total read abundance. We selected the five most abundant for fold‐change analysis. For each species, we calculated relative abundance per sample by dividing its read count by the total animal reads. Samples were paired by collection time and place, and the fold change for each pair was calculated as the ratio of the relative abundance with the blocker to that without the blocker. For each species, we recorded the number of pairs in which it was detected, the mean relative abundance with and without the blocker, the mean fold change across pairs in which the species was present in at least one condition, and the number of pairs in which the species became completely absent when the blocker was used. We also checked, for both prey groups and individual species, if any taxa were detected only in one condition (Blocker or No Blocker) across all paired fecal samples to identify taxa that might have been entirely gained or lost due to blocking, rather than just showing a change in relative abundance.

The fecal dataset that was collected in the field (consisting of 12 paired samples) was processed using the same DADA2 pipeline and reference database. Since taxonomic information for the extra pairs was available only at the species level, prey richness, diversity, and community composition were calculated using a species‐level community matrix to maintain consistency across the combined dataset. Before the analysis, any reads assigned to *Homo sapiens* that were identified as contamination were excluded.

## 3 Results

### 3.1 DNA Extraction Kits Test

**FIGURE 2:**
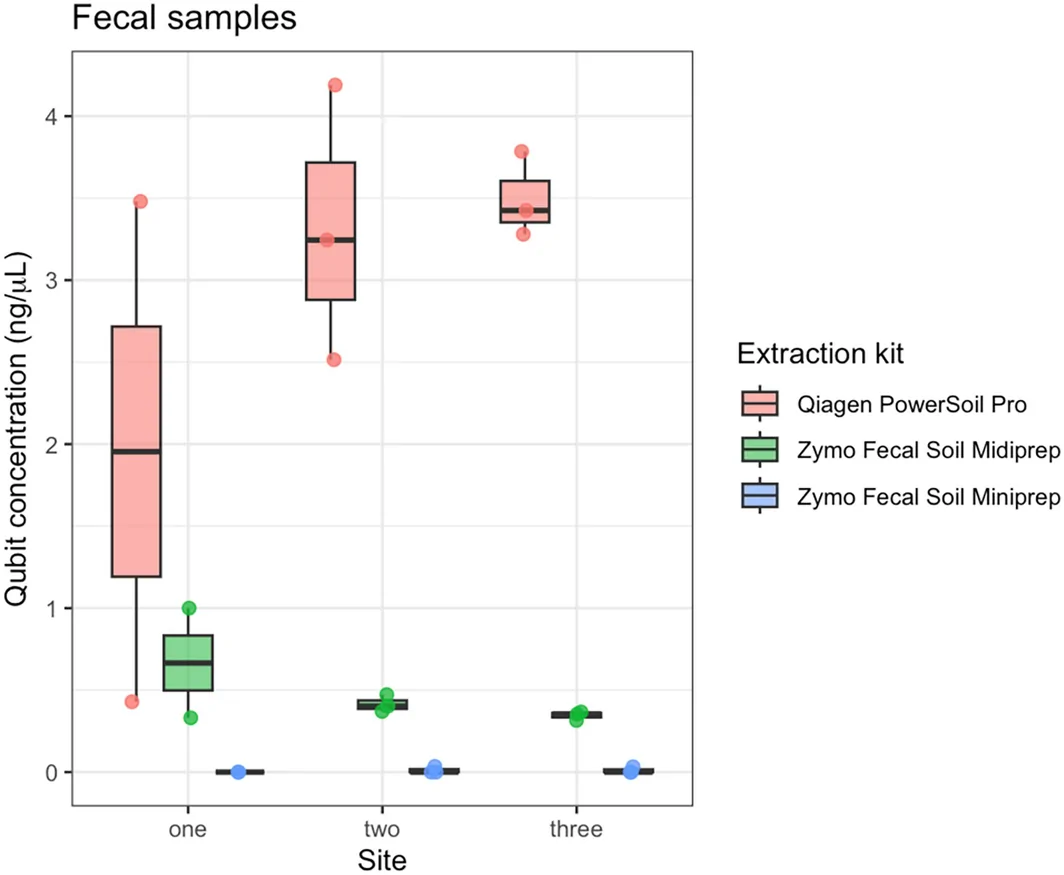
Boxplot showing extracted DNA concentration (ng/μL) from the fecal samples collected from Cape Cod, Massachusetts, by extraction kit (pink = Qiagen PowerSoil Pro kit, green = Zymo Quick‐DNA Fecal/Soil Microbe Midi Kit, blue = Zymo Quick‐DNA Fecal/Soil Microbe Mini Kit). There was a substantial, significant difference in the effects of extraction kits on DNA concentration (*p* = 2.04 × 10^−8^).

**FIGURE 3:**
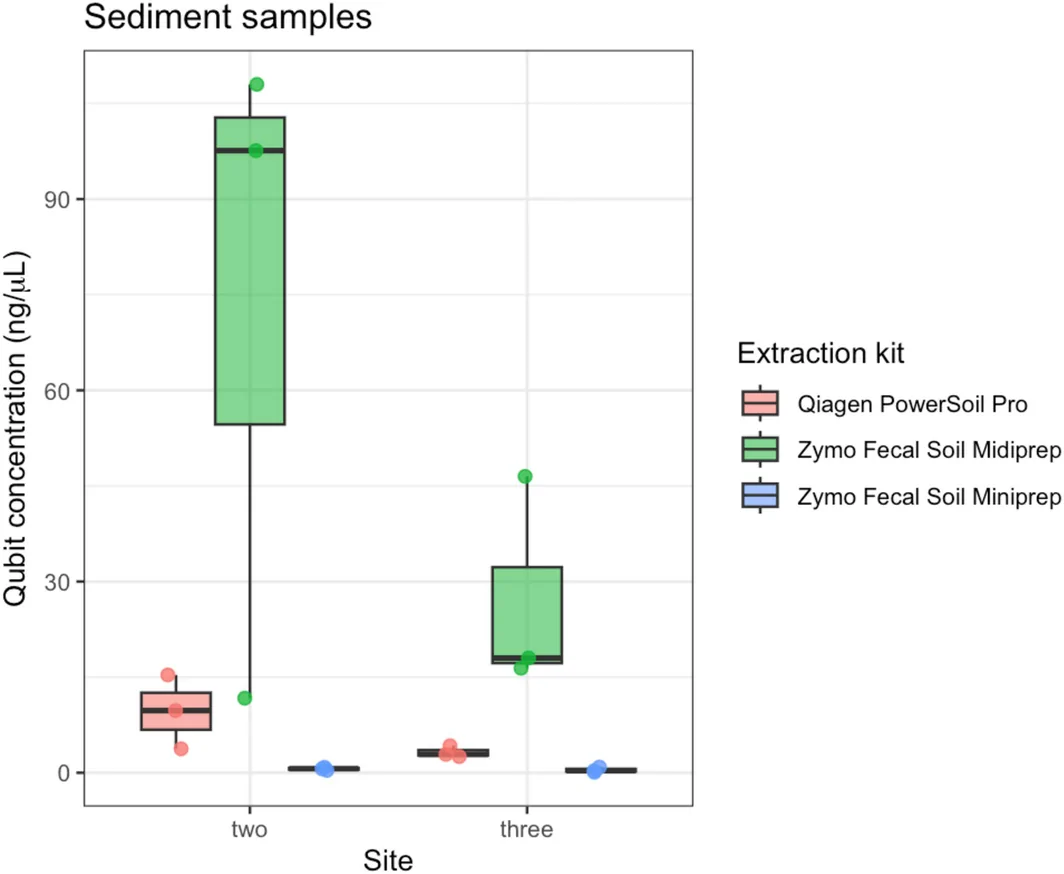
Boxplot showing the extracted DNA concentration (ng/μL) from the sediment samples collected from Cape Cod, Massachusetts, by extraction kit (pink = Qiagen PowerSoil Pro kit, green = Zymo Quick‐DNA Fecal/Soil Microbe Midi Kit, blue = Zymo Quick‐DNA Fecal/Soil Microbe Mini Kit). There was a significant difference in the effect of the extraction kits on the DNA concentrations (*p* = 0.006).

A total of 18 samples (9 fecal and 9 sediment) were successfully extracted with the three different extraction kits: the Qiagen PowerSoil Pro Kit, the Zymo Midi Kit, and the Zymo Mini Kit, following the manufacturer's instructions. To account for repeated extractions from the sample, a linear mixed model with sample/site as a random effect was fitted to the DNA concentrations. The extraction kits had significant effects on DNA yield in both fecal (*F* = 46.2, *p* = 2.04 × 10^−8^) and sediment (*F* = 7.54, *p* = 0.006) samples (Figures 2 and 3, respectively). The Qiagen PowerSoil Pro extraction kit yielded more DNA from the fecal samples (Figure 2, *p* = 2.04 × 10^−8^), with DNA concentrations ranging from 2 to 4 ng/μL. In comparison, the Zymo Midi extraction kit yielded more DNA from the sediment samples (Figure 3, *p* = 0.00747), with DNA concentrations ranging from 15 to 90 ng/μL.

For the extraction‐kit metabarcoding comparison (Qiagen PowerSoil Pro vs. Zymo Midi, *n* = 5 samples), paired *t*‐tests on richness (*t* = −1.09, *p* = 0.336), Shannon diversity (*t* = −0.50, *p* = 0.644), and Chao1 (*t* = −1.19, *p* = 0.298) were all nonsignificant. We also ran PERMANOVA (*R* ^2^ = 0.060, *p* = 0.063) and beta‐dispersion (*p* = 0.529) for composition, both of which were nonsignificant. Metabarcoding of the 5 samples extracted with the Qiagen PowerSoil Pro and Zymo Midi kits yielded 2006 unique ASVs, with an average of 468.3 ± 119.6 SD ASV richness per sample. Read depth for these 10 samples ranged from 46,330 to 65,532 (mean 53,314 ± 5897), and accumulation curves leveled off at 10,000 for each sample (Figure S1), supporting adequate sequencing depth.

### 3.2 Blocking Primer Validation

In the lab tests, one of the candidate blocking primers (F2S: 5′‐CAGTATACCCCCCACTCGCTGG/3SpC3/‐3′) reduced the Red Knot DNA amplification while not significantly affecting the amplification of the invertebrates' DNA as determined visually via an agarose gel. In addition to the gel‐based test, an in silico sequence alignment found no exact match of 8 or more matching bases between the blocking primer and the detected prey taxa tested, with the best overall identity across the full 22 bp primer ranging from 50% to 54.5%, including for *Limulus polyphemus* , one of our major prey findings. This indicates that the blocking primer does not physically bind to these five prey taxa, making direct sequence‐based blocking an unlikely explanation for any suppression of their detection.

**FIGURE 4:**
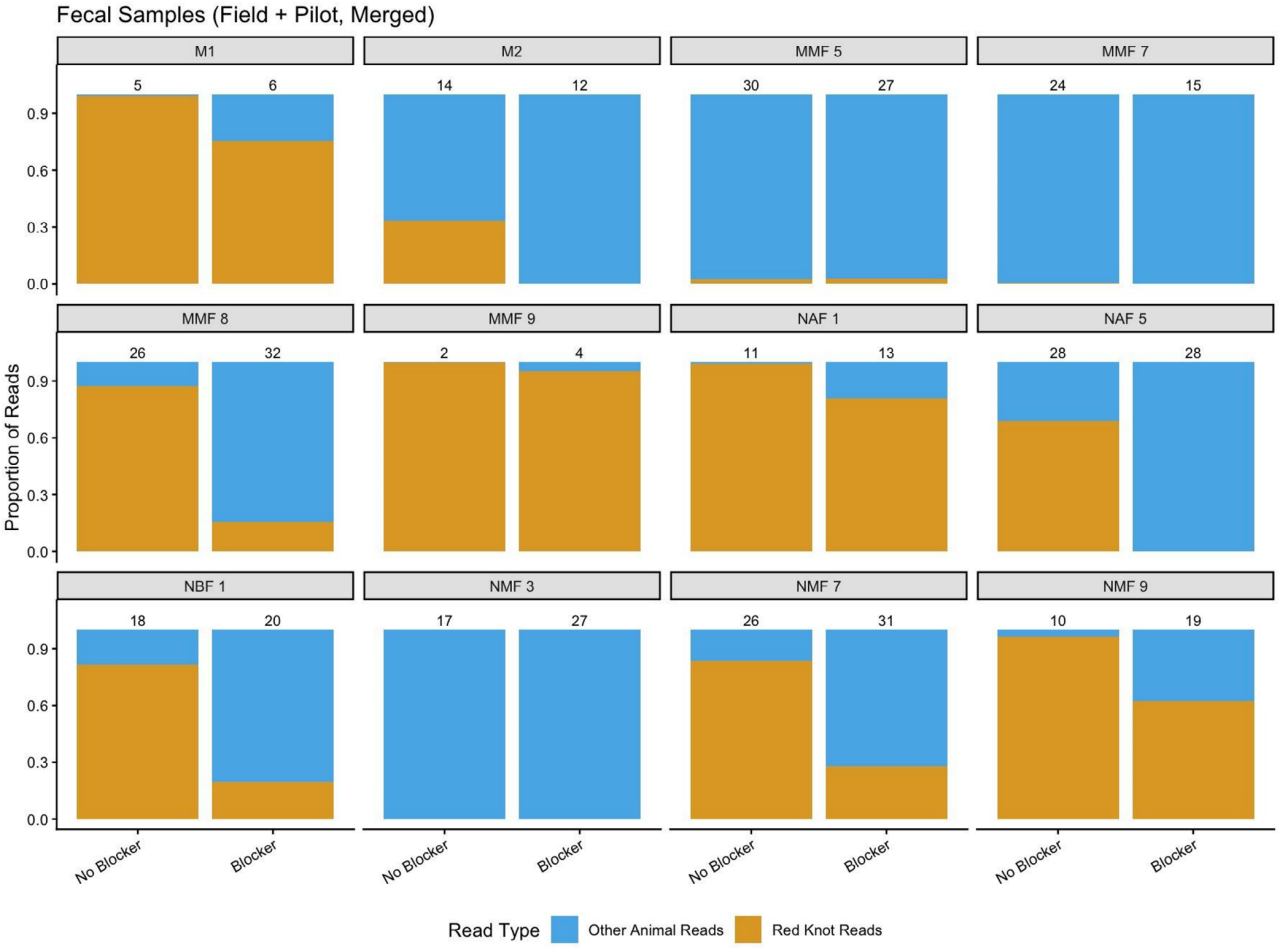
This bar plot shows the proportions of Red Knot DNA in the fecal samples with and without the blocking primer. The *x*‐axis shows the different treatments given to the fecal samples, while the *y*‐axis shows the proportions of total read counts in the samples. The letters represent the site names, and the numbers represent the number of prey species detected at each site. This plot was generated in R version 4.3.2.

To more thoroughly assess blocking primer performance at field scale, we sequenced field fecal samples (*n* = 12 paired samples) and sediment samples (*n* = 7 paired samples), collecting samples both with and without the blocking primer. In fecal samples that contained host DNA (*n* = 11 of the 12 pairs; one pair had no detectable host DNA rather than the Short‐billed Dowitcher under either condition), the mean proportion of host DNA dropped significantly from 68.3% in the absence of the blocking primer to 34.6% when the primer was used (paired *t* = 4.11, df = 10, *p* = 0.002; Wilcoxon signed‐rank *p* = 0.003) (Figure 4). The greatest reduction in host DNA amplification in a single sample was observed in sample NAF 5, where the proportion of host DNA fell from 68.8% of reads without the blocker to 0.2% with the blocker. Prey species richness averaged 12.8 ± 2.1 species (mean ± SE) when the blocking primer was used, as opposed to 11.1 ± 2.0 species without it (mean difference = 1.75 species), a difference that was significant according to the paired *t*‐test (*t* = 2.43, df = 11, *p* = 0.033) but not according to the nonparametric Wilcoxon signed‐rank test (*p* = 0.054). Shannon diversity (paired *t* = −0.69, df = 11, *p* = 0.507) and the overall community composition (PERMANOVA *R* ^2^ = 0.009, *p* = 0.774; beta‐dispersion *p* = 0.886) remained unchanged. Sediment pairs showed no significant difference in Shannon diversity (*p* = 0.615) or community composition (PERMANOVA *R* ^2^ = 0.013, *p* = 0.234). Richness was also not significantly different between conditions, though it fell just short of conventional significance (*t* = 2.41, df = 6, *p* = 0.053). This suggests that the blocker's effect on sediment prey detection was at most modest.

To determine whether the blocking primer's effect on prey detection was consistent across species or varied by species, we ranked all prey species by total read abundance. Then we examined the fold change in relative abundance (comparing blocked to unblocked conditions) for the five most abundant species. The effect was found to be species‐dependent and not uniform. *Limulus polyphemus* (the horseshoe crab), a major prey resource in this system, showed a 1.83‐fold increase in relative abundance when detected in 4 of the 12 paired samples, with no cases of complete dropout. *Littorina littorea* exhibited the greatest increase, at 2.08‐fold, in the two pairs in which it was detected. *Chiridotea coeca* showed a more modest 1.41‐fold increase over 5 pairs, although it reached zero reads in one of them. On the other hand, both *Cassidinidea lunifrons* and *Alitta virens* displayed net decreases under blocking (0.46‐fold and 0.53‐fold, respectively), each detected in only 2 pairs, with one pair per species showing complete suppression. The detection counts for these species ranged from 2 to 5 across the 12 paired samples; therefore, the fold‐change estimates based on only 2 pairs should be interpreted with caution.

### 3.3 Red Knot Diet Field Survey

A total of 108 field fecal and sediment samples were collected for the field survey: 32 were individual fecal samples, and 76 were sediment samples. Sediment samples were pooled by site and collection date into 20 composite samples, which included 3 from Little Sipson, 4 from Minimoy, 3 from Nauset, 3 from North Beach, 4 from North Monomoy, and 3 from Tern Island. Of the 52 sequenced samples (32 fecal and 20 pooled sediment samples), 15 contained host DNA ASVs, all of which were fecal samples, and approximately 28% of the total reads were assigned to Red Knots. No amplification was observed in the negative control, but amplification of mock species was observed in the positive controls.

A total of 3,256,923 reads were obtained after quality filtering, yielding 4717 unique ASVs, of which approximately 72% of ASVs were taxonomically identified, mainly in the classes Bivalvia, Arachnida, Malacostraca, Merostomata, Clitellata, Gastropoda, Polychaeta, and Insecta. Per‐sample read counts averaged 62,633 ± 12,486 SD (range: 26,239–85,398 reads); no samples were excluded due to insufficient read depth. None of the 52 sequenced samples came back at zero.

In the fecal samples, the classes Merostomata and Malacostraca were abundant, as expected, since they are known prey of Red Knots. Aside from these major prey items detected, other commonly detected taxa are associated with the foraging habitats of these Red Knots. These included the Bivalvia and Gastropoda, which are also recorded. Benthic invertebrates were also detected in the fecal samples, including Polychaeta and Clitellata; these are primarily associated with intertidal sediments. The classes Insecta and Arachnida were also detected, likely reflecting incidental ingestion. No unexpected classes of prey items were detected, and the detected taxonomic groups aligned with the known feeding ecology of the Red Knots.

For the fecal samples, PERMANOVA indicated a significant difference among sites (*R* ^2^ = 0.134, *p* = 0.009). Beta‐dispersion was also significant (*p* = 0.001), due to variability in site sample sizes (e.g., North Beach *n* = 2 vs. North Monomoy *n* = 14). For the sediment samples, PERMANOVA returned *R* ^2^ = 0.351, *p* = 0.001, and dispersion was not a significant factor (*p* = 0.914), indicating a real difference in composition between sites. These tests on the sediment samples were conducted using the 20 pooled composite samples as the unit of replication, consistent with their sequencing, rather than treating them as independent samples. However, the overall comparison between fecal and sediment samples remained significant (PERMANOVA *R* ^2^ = 0.038, *p* = 0.001); the effect was small and attributable to differences in variability between groups rather than a true difference in composition (*p* = 0.029).

## 4 Discussion

In this study, we validated a blocking primer that prevents amplification of Red Knot ( *Calidris canutus* ) DNA while increasing amplification of DNA from other invertebrates. The Red Knot host‐specific blocking primer, designed for the Leray primer sets, notably increased our capacity to detect prey items in fecal samples and, where applicable, environmental samples collected from the Red Knot and its foraging environment. In the absence of blocking primers, host DNA dominated most samples, as indicated by the sequence data (Table S1), comprising up to 99.3% of total reads in some samples. This resulted in a substantially masked dietary signal, as host DNA reads vastly outnumbered prey reads in most unblocked samples. In contrast, sediment samples contained no detectable Red Knot DNA. While this precludes assessment of host DNA suppression specifically, we directly tested whether the blocking primer affected nontarget taxa in sediment samples; ASV richness, Shannon diversity, and community composition did not differ significantly between blocked and unblocked sediment samples (see Section 3.2), indicating no detectable effect of the blocking primer on the sediment prey community. The result is consistent with the existing literature, which shows that host DNA frequently predominates in noninvasive dietary samples, particularly when universal primers such as the Leray COI primer set are used, thereby concealing dietary signals (Deagle et al. 2009).

On the other hand, applying blocking primers to the samples (Figure 4) significantly reduced host DNA sequence reads, thereby increasing the relative abundances of the various prey sequences. We then examined how the abundance of each prey group changed across all 12 samples tested.

The Merostomata and the Malacostraca were the most common prey taxa found in the fecal samples (Section 3.3). Earlier studies demonstrated that the use of blocking primers can reduce the amplification of host DNA and enhance the detection of less abundant prey taxa. Blocking primers have proven effective in gut‐content studies of other predators (Vestheim and Jarman 2008) and insect‐eating birds (Jedlicka et al. 2017), but their application to shorebirds has not been demonstrated. Horseshoe crab ( *Limulus polyphemus* ) eggs are an essential food resource for migrating Red Knots, making detection of this prey a key test of the method's suitability for shorebird feeding ecology. As reported in Section 3.2, *Limulus polyphemus* detection was robust to the blocking primer treatment in 4 of the 12 paired fecal samples in which it was detected, with no cases of complete suppression. This persistence demonstrates that blocking primers can be applied to shorebird fecal DNA metabarcoding without erasing signals from a prey species central to the Red Knot diet. By contrast, both *Cassidinidea lunifrons* and *Alitta virens* exhibited net decreases in relative abundance when blocking primers were used. This indicates that suppression, when it occurs, is specific to individual species rather than affecting entire taxonomic groups. We recommend confirmatory no‐blocker sequencing for studies targeting these species in shorebird feeding‐ecology research.

In the fecal samples, which represent what the Red Knots potentially fed on, there was an increased detection of prey items across multiple taxa, including copepods, rotifers, nematodes, and mollusks, when the Red Knot DNA in the samples was blocked. The sediment samples, which represent the foraging environment, also showed similar prey items. This combined analysis of fecal and environmental samples strengthens inference about prey availability and dietary intake in complex coastal systems (Baker et al. 2004). Overall, the use of blocking primers and the Leray primers was effective for amplifying prey items across a broad array of taxa while suppressing amplification of host DNA. This finding supports those of Leray et al. (2013), who demonstrated the primers' versatility across marine taxa. Although the pilot study involved only 12 samples, the consistent findings indicate that the blocking primer is a robust tool for dietary assessment, even with a small sample size.

We also tested Red Knot fecal and sediment samples using three different extraction kits, following the manufacturer's instructions, to assess their performance in yielding DNA. The Qiagen PowerSoil Pro kit yielded the most DNA from the fecal samples (Figure 2) as compared to the other extraction kits. In contrast, the Zymo Midi kit yielded the most DNA from the sediment samples (Figure 3) as compared to the other extraction kits. The Zymo Mini kit yielded the lowest DNA amount across both sediment and fecal samples. These results suggest that the extraction protocol, especially in diet analysis, should be tailored to the sample type, as no single extraction kit performs optimally across all sample types. However, DNA concentration was not associated with ASV richness, diversity, or community composition.

Nevertheless, there was no significant difference in species richness or community composition between the PowerSoil Pro and Zymo Midi kits. Using nonoptimized extraction kits can reduce both DNA yield and the number of targeted taxa. This is most common when working with low‐concentration or degraded DNA sources. PCR inhibitors are predominantly present in fecal samples, and the Qiagen PowerSoil Pro kit efficiently removes them. This result was also observed in a study by Hou et al. (2021), which compared three extraction methods for bird feces and found that Qiagen performed best for alpha diversity. The Zymo kit can handle larger input volumes, so it extracted more DNA from the sediment samples. However, in the remainder of the study, the PowerSoil Pro kit was used to extract DNA from both fecal and sediment samples.

**FIGURE 5:**
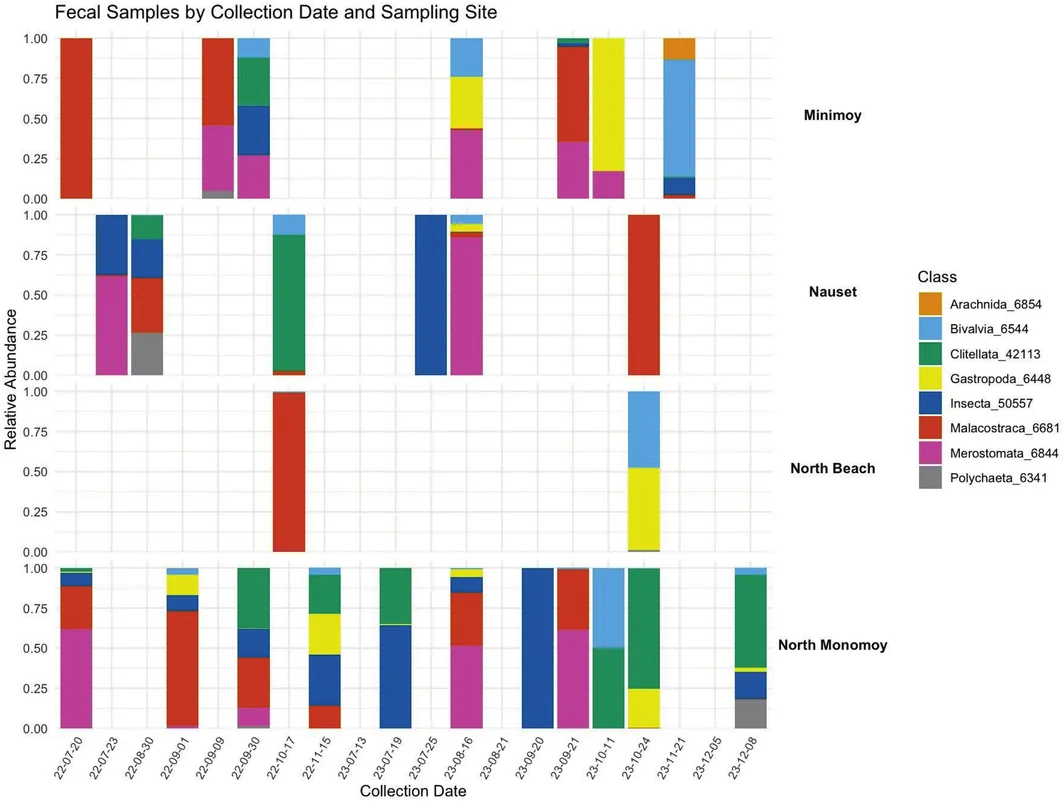
Relative abundance of prey classes in fecal samples by collection date and sampling site. Bar plot for fecal samples generated in R version 4.3.2.

**FIGURE 6:**
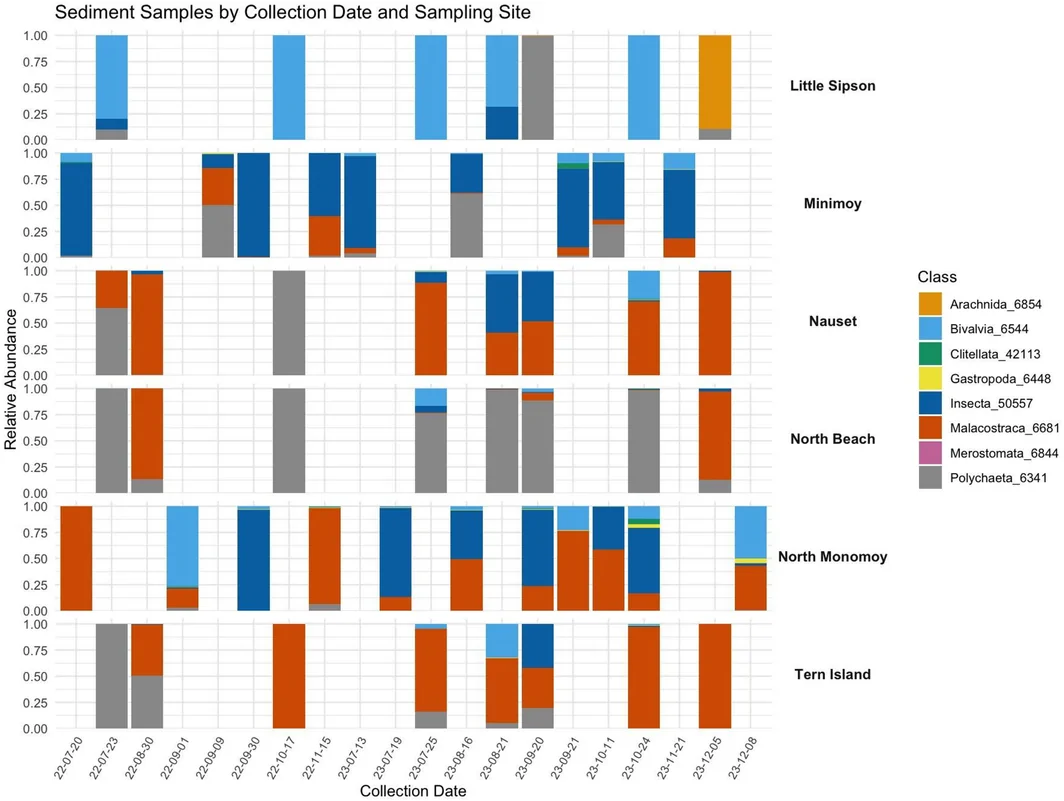
Relative abundance of prey classes in pooled sediment composites by collection date and sampling site. Bar plot for sediment samples generated in R version 4.3.2.

In all 108 samples (which included 32 individual fecal samples and 76 sediment samples pooled to make 20 composite samples), the blocking primer was used on every sample, and in the samples in which host DNA was present, about 28% of the reads were attributed to Red Knot DNA. To directly measure the degree of host DNA reduction under field conditions, several field‐collected fecal and sediment samples were also sequenced with and without the blocking primer (12 fecal pairs and 7 sediment pairs). In the case of the fecal pairs in which host DNA was found (*n* = 11 of the 12 pairs; one pair had no detectable host DNA under either condition), the mean proportion of host DNA fell significantly from 68.3% in the absence of the blocker to 34.6% in the presence of the blocker (paired *t* = 4.11, df = 10, *p* = 0.002), together with a small increase in the number of prey species (mean + 1.75 species) which was significant according to the paired *t*‐test (*p* = 0.033) but not by the nonparametric Wilcoxon test (*p* = 0.054), thus directly demonstrating the effectiveness of the blocking primer at the field level. There was no amplification in the negative controls; amplification was observed in the positive controls, confirming both the specificity of the blocking primers and the efficiency of the PCR reactions. Although the large sample size indicates that this method can handle large ecological datasets, its specificity for prey DNA is more complicated. The primer sequences did not match five major prey groups, such as *Limulus polyphemus* , and the addition of the blocker increased the number of prey species detected without altering the overall composition of the prey community (Section 3.2). However, when the five most abundant prey species were examined individually, *Cassidinidea lunifrons* and *Alitta virens* showed a net decrease in relative abundance under blocking, indicating that suppression, when it occurs, is specific to individual species rather than affecting all taxonomic groups equally. For this reason, we advise that any future studies concentrating on these species should also sequence the samples without the blocker to verify the results. Figures 5 and 6 show apparent spatiotemporal variation in the relative abundance of prey DNA sequence classes in fecal and sediment samples collected from various sites (Nauset, Minimoy, North Monomoy, North Beach, Tern Island, and Little Sipson) and at multiple time points. The different taxa of the prey items identified included the Bivalvia, Arachnida, Malacostraca, Merostomata, Clitellata, Gastropoda, Polychaeta, and Insecta. The most significant abundances of copepods and mollusks align with the findings of Colwell and Landrum (1993), who reported that these taxa dominate benthic invertebrate communities in intertidal coastal habitats and represent readily available prey for shorebirds.

Several limitations of this study should be acknowledged. The sampling was limited to the southbound migration season, thereby limiting inferences about dietary variation across the other seasons of the migration cycle. However, this method can be applied to other seasons as well. The metabarcoding reflected the relative sequence abundance of the prey taxa, not their biomass, and could not differentiate between life stages of the prey taxa; for example, horseshoe crab eggs versus larvae. Prey taxa identified in the sediment samples describe the environment, not direct consumption. The blocking‐primer suppression was not uniform across individual samples. In one fecal field replicate (Figure 4), the host DNA proportion changed negligibly between blocked and unblocked conditions despite adequate sequencing depth, indicating that the blocking primer did not suppress host amplification in that individual sample. This is a recognized limitation of blocking‐primer approaches and does not undermine the significant population‐level suppression effect demonstrated across the full paired dataset. In the case of another fecal sample (MMF 7), the number of prey species fell when the blocker was used (24 species in the absence of the blocker and 15 in its presence), the total prey read depth being similar in the two conditions (28,467 versus 27,925 reads), which shows that the change in community evenness was real and not due to a sequencing artifact.

Additionally, sediment samples typically contain DNA from nontarget organisms, making the selective amplification of prey items (using blocking primers) extremely valuable. When well‐designed and validated, blocking primers mask host DNA rather than unwanted prey, allowing signals from true prey taxa to be readily detected (Taberlet et al. 2012); however, poorly designed or insufficiently tested blocking primers can inadvertently suppress prey taxa as well, underscoring the importance of the sequence‐alignment and prey‐community testing conducted here (Sections 3.2 and 3.3). Despite these limitations, the consistency of the results indicates that the blocking primer assay is effective and scalable.

Beyond methodological validation, our results also provided ecologically meaningful insights into Red Knot feeding ecology that align with the historical literature. The dominance of copepods, crustaceans, mollusks, and other benthic invertebrates among detected prey taxa indicates that the Red Knot is an opportunistic forager when necessary, particularly during migration and stopover periods (Baker et al. 2004; Colwell and Landrum 1993). The detection of the Merostomata taxa suggests the consumption of horseshoe crab eggs or larvae during their southbound migration. This is striking because the consumption of energy‐rich horseshoe crab eggs by shorebirds is typically documented during northbound migration. The spawning of horseshoe crab eggs is largely reduced from July onward, and so shorebirds are thought to feed heavily on benthic invertebrates during their southbound migration (MDMF 2023). The detection of horseshoe crab eggs in both sediment and fecal samples during the sampling period indicates that horseshoe crab eggs remain available in the environment and are incorporated into Red Knot diets during southbound migration, despite reduced spawning activity compared to the northbound (spring) migration period. The *rufa* Red Knots are listed as threatened, underscoring the importance of maintaining this key prey source during both northbound and southbound migration. This result is significant for informing conservation efforts and management strategies for horseshoe crabs, given ongoing harvest pressure on populations in Massachusetts and other regions. The dietary metabarcoding approach used here provides a noninvasive means of detecting prey and can complement traditional methods. Although it also identifies taxa that may be accidentally consumed rather than targeted prey, this could be heavily reflected in prey identification.

In summary, this study reaffirms the importance of combining a Red Knot blocking primer with the Leray COI primer sets to determine prey items in fecal and sediment samples, making the method a powerful tool for dietary assessment. Future studies could incorporate seasonal sampling, as the samples in this study were collected in the fall, thereby enabling tracking and understanding of dietary changes over time and further validating the blocking primer used. This will refine our understanding of migratory shorebird feeding ecology and inform conservation management and strategies.

## Author Contributions

**Christiana K. Teye:** conceptualization (equal), writing – original draft (equal), writing – review and editing (equal). **Erin K. Grey:** conceptualization (equal), writing – original draft (equal). **Alan Kneidel:** conceptualization (equal), writing – original draft (equal). **Stephen Brown:** conceptualization (equal), writing – original draft (equal). **Liana DiNunzio:** conceptualization (equal), writing – original draft (equal).

## Funding

This work was supported by U.S. Fish and Wildlife Service under a Recovery Challenge Grant (F22AC00880‐00) and Maine Center for Genetics in the Environment National Science Foundation (OIA‐1849227).

## Conflicts of Interest

The authors declare no conflicts of interest.

## Supporting information


**Table S1:** Summary of Red Knot and other animal taxa across samples in the Blocker Test.
**Figure S1:** Rarefaction (ASV accumulation) curves for the extraction‐kit test samples. This was generated in R version 4.3.2.

## Data Availability

The raw sequence data will be deposited in the NCBI Sequence Read Archive. The entire bioinformatic workflow for each run, including parameter values and a summary of reads at each step for each sample, is available at https://github.com/egreyavis/REKN_blocker_dvlpmt.git.

## References

[ece374283-bib-0001] Andrews, S. 2010. “FastQC: A Quality Control Tool for High‐Throughput Sequence Data.” http://www.bioinformatics.babraham.ac.uk/projects/fastqc.

[ece374283-bib-0002] Baker, A. J. , P. M. González , T. Piersma , et al. 2004. “Rapid Population Decline in Red Knots: Fitness Consequences of Decreased Refueling Rates and Late Arrival in Delaware Bay.” Proceedings of the Royal Society B: Biological Sciences 271, no. 1541: 875–882. 10.1098/rspb.2003.2663. PMC1691665 15255108

[ece374283-bib-0003] Barrett, R. T. , K. C. Camphuysen , T. Anker‐Nilssen , et al. 2007. “Diet Studies of Seabirds: A Review and Recommendations.” ICES Journal of Marine Science 64, no. 9: 1675–1691. 10.1093/icesjms/fsm152.

[ece374283-bib-0059] Callahan, B. J. , P. J. McMurdie , M. J. Rosen , A. W. Han , A. J. A. Johnson , and S. P. Holmes . 2016. “DADA2: High‐Resolution Sample Inference From Illumina Amplicon Data.” Nature Methods 13, no. 7: 581–583. 27214047 10.1038/nmeth.3869 PMC4927377

[ece374283-bib-0004] Camacho, C. , G. Coulouris , V. Avagyan , et al. 2009. “BLAST+: Architecture and Applications.” BMC Bioinformatics 10: 421. 20003500 10.1186/1471-2105-10-421 PMC2803857

[ece374283-bib-0005] Chan, Y. C. , H. B. Peng , Y. X. Han , et al. 2019. “Conserving Unprotected Important Coastal Habitats in the Yellow Sea: Shorebird Occurrence, Distribution, and Food Resources at Lianyungang.” Global Ecology and Conservation 20: e00724.

[ece374283-bib-0007] Colwell, M. A. , and S. L. Landrum . 1993. “Nonrandom Shorebird Distribution and Fine‐Scale Variation in Prey Abundance.” Condor 95, no. 1: 94–103.

[ece374283-bib-0008] Davis, B. Y. , Y. Shah Esmaeili , H. Goldspiel , et al. 2024. “Reference Sequence Library Resources – Maine‐eDNA (4.0) [Data Set].” *Zenodo*. 10.5281/zenodo.14232801.

[ece374283-bib-0009] Deagle, B. E. , N. J. Gales , K. Evans , et al. 2007. “Studying Seabird Diet Through Genetic Analysis of Feces: A Case Study on Macaroni Penguins ( *Eudyptes chrysolophus* ).” PLoS One 2, no. 9: e831. 17786203 10.1371/journal.pone.0000831 PMC1959119

[ece374283-bib-0010] Deagle, B. E. , R. Kirkwood , and S. N. Jarman . 2009. “Analysis of Australian Fur Seal Diet by Pyrosequencing Prey DNA in Feces.” Molecular Ecology 18, no. 9: 2022–2038. 10.1111/j.1365-294X.2009.04158.x. 19317847

[ece374283-bib-0011] Deiner, K. , H. M. Bik , E. Mächler , et al. 2017. “Environmental DNA Metabarcoding: Transforming How We Survey Animal and Plant Communities.” Molecular Ecology 26, no. 21: 5872–5895. 28921802 10.1111/mec.14350

[ece374283-bib-0013] Duijns, S. , L. J. Niles , A. Dey , et al. 2017. “Body Condition Explains the Migratory Performance of a Long‐Distance Migrant.” Proceedings of the Royal Society B: Biological Sciences 284, no. 1866: 20171374. 10.1098/rspb.2017.1374 PMC5698639 29093218

[ece374283-bib-0014] Edenfield, L. , and D. M. Edenfield . 2024. “Fish and Wildlife Service.”

[ece374283-bib-0015] Giebner, H. , K. Langen , S. J. Bourlat , et al. 2020. “Comparing Diversity Levels in Environmental Samples Using DNA Sequence Capture and Metabarcoding Approaches Targeting the 18S and COI Genes.” Molecular Ecology Resources 20, no. 5: 1333–1345. 32462738 10.1111/1755-0998.13201

[ece374283-bib-0016] Goldberg, C. S. , C. R. Turner , K. Deiner , et al. 2016. “Critical Considerations for Applying Environmental DNA Methods to Detect Aquatic Species.” Methods in Ecology and Evolution 7, no. 11: 1299–1307.

[ece374283-bib-0017] Goss‐Custard, J. D. , R. A. Stillman , A. D. West , et al. 2006. “Intake Rates and the Functional Response in Shorebirds: The Effects of Interference and Abundance.” Ecological Monographs 76, no. 2: 259–281. 10.1890/0012-9615(2006)076[0259:IRATFR]2.0.CO;2.

[ece374283-bib-0060] Hamilton, D. J. , A. W. Diamond , and P. G. Wells . 2006. “Shorebirds, Snails, and the Amphipod (*Corophium volutator*) in the Upper Bay of Fundy: Top–Down vs. Bottom–Up Factors, and the Influence of Compensatory Interactions on Mudflat Ecology.” Hydrobiologia 567, no. 1: 285–306.

[ece374283-bib-0019] Harrington, B. A. , N. P. Hill , and B. Nikula . 2010. “Changing Use of Migration Staging Areas by Red Knots: A Historical Perspective From Massachusetts.” Waterbirds 33, no. 2: 188–192. 10.1675/063.033.0207.

[ece374283-bib-0020] Harrington, B. A. , S. Koch , L. K. Niles , and K. Kalasz . 2010. “Red Knots With Different Winter Destinations: Differential Use of an Autumn Stopover Area.” Waterbirds 33, no. 3: 357–363. 10.1675/063.033.0312.

[ece374283-bib-0022] Hou, X. , S. Pan , Z. Lin , J. Xu , and X. Zhan . 2021. “Performance Comparison of Different Microbial DNA Extraction Methods on Bird Feces.” Avian Research 12, no. 1: 19.

[ece374283-bib-0023] Iwamura, T. , H. P. Possingham , I. Chadès , et al. 2013. “Migratory Connectivity Magnifies the Consequences of Habitat Loss From Sea‐Level Rise for Shorebird Populations.” Proceedings of the Royal Society B: Biological Sciences 280, no. 1761: 20130325. 10.1098/rspb.2013.0325 PMC3652437 23760637

[ece374283-bib-0024] Jedlicka, J. A. , A. T. E. Vo , and R. P. Almeida . 2017. “Molecular Scatology and High‐Throughput Sequencing Reveal Predominantly Herbivorous Insects in the Diets of Adult and Nestling Western Bluebirds ( *Sialia mexicana* ) in California Vineyards.” Auk: Ornithological Advances 134, no. 1: 116–127.

[ece374283-bib-0025] Leray, M. , N. Knowlton , and R. J. Machida . 2022. “MIDORI2: A Collection of Quality‐Controlled, Preformatted, and Regularly Updated Reference Databases for Taxonomic Assignment of Eukaryotic Mitochondrial Sequences.” Environmental DNA 4, no. 4: 894–907.

[ece374283-bib-0026] Leray, M. , J. Y. Yang , C. P. Meyer , et al. 2013. “A New Versatile Primer Set Targeting a Short Fragment of the Mitochondrial COI Region for Metabarcoding Metazoan Diversity: Application for Characterizing Coral Reef Fish Gut Contents.” Frontiers in Zoology 10, no. 1: 34. 10.1186/1742-9994-10-34. 23767809 PMC3686579

[ece374283-bib-0027] MacDonald, A. J. , P. A. Smith , C. A. Friis , J. E. Lyons , Y. Aubry , and E. Nol . 2021. “Stopover Ecology of Red Knots in Southwestern James Bay During Southbound Migration.” Journal of Wildlife Management 85, no. 5: 932–944.

[ece374283-bib-0028] Martin, M. 2011. “Cutadapt Removes Adapter Sequences From High‐Throughput Sequencing Reads.” EMBnet.Journal 17, no. 1: 10–12. https://journal.embnet.org/index.php/embnetjournal/article/view/200.

[ece374283-bib-0029] Massachusetts Division of Marine Fisheries . 2023. Observing Horseshoe Crabs in Massachusetts. Commonwealth of Massachusetts. https://www.mass.gov/info‐details/observing‐horseshoe‐crabs‐in‐massachusetts.

[ece374283-bib-0030] McMurdie, P. J. , and S. Holmes . 2013. “phyloseq: An R Package for Reproducible Interactive Analysis and Graphics of Microbiome Census Data.” PLoS One 8, no. 4: e61217. 23630581 10.1371/journal.pone.0061217 PMC3632530

[ece374283-bib-0033] Niles, L. J. , J. O. A. N. N. A. Burger , R. R. Porter , et al. 2010. “First Results From Light‐Level Geolocators Used to Track Red Knots in the Western Hemisphere Reveal Rapid, Long Intercontinental Flights and New Details on Migration Pathways.” Wader Study Group Bulletin 117, no. 2: 123–130.

[ece374283-bib-0034] Oksanen, J. , G. Simpson , F. Blanchet , et al. 2025. “vegan: Community Ecology Package.” R Package Version 2.8‐0. https://vegandevs.github.io/vegan/.

[ece374283-bib-0057] Pebesma, E. 2018. “Simple Features for R: Standardized Support for Spatial Vector Data.” R Journal 10: 439–446.

[ece374283-bib-0035] Piersma, T. 2003. ““Coastal” Versus “Inland” Shorebird Species: Interlinked Fundamental Dichotomies Between Their Life and Demographic Histories?” Wader Study Group Bulletin 100: 5–9.

[ece374283-bib-0036] Piersma, T. , A. Koolhaas , and A. Dekinga . 1993. “Interactions Between Stomach Structure and Diet Choice in Shorebirds.” Auk 110: 552–564.

[ece374283-bib-0037] Piersma, T. , T. Lok , Y. Chen , et al. 2016. “Simultaneous Declines in Summer Survival of Three Shorebird Species Signal a Flyway at Risk.” Journal of Applied Ecology 53, no. 2: 479–490.

[ece374283-bib-0038] Pompanon, F. , B. E. Deagle , W. O. C. Symondson , D. S. Brown , S. N. Jarman , and P. Taberlet . 2012. “Who Is Eating What: Diet Assessment Using Next‐Generation Sequencing.” Molecular Ecology 21, no. 8: 1931–1950. 10.1111/j.1365-294X.2011.05403.x. 22171763

[ece374283-bib-0042] Ruan, H. T. , R. L. Wang , H. T. Li , et al. 2022. “Effects of Sampling Strategies and DNA Extraction Methods on eDNA Metabarcoding: A Case Study of Estuarine Fish Diversity Monitoring.” Zoological Research 43, no. 2: 192. 35084125 10.24272/j.issn.2095-8137.2021.331 PMC8920846

[ece374283-bib-0044] Smith, S. M. 2015. “Vegetation Change in Salt Marshes of Cape Cod National Seashore (Massachusetts, USA) Between 1984 and 2013.” Wetlands 35, no. 1: 127–136. 10.1007/s13157-014-0601-7.

[ece374283-bib-0058] South, A. 2017. “rnaturalearth: World Map Data From Natural Earth.” R Package Version 0.1.0.

[ece374283-bib-0045] Stoeck, T. , D. Bass , M. Nebel , et al. 2010. “Multiple‐Marker Parallel Tag Environmental DNA Sequencing Reveals a Highly Complex Eukaryotic Community in Anoxic Marine Waters.” Molecular Ecology 19: 21–31. 20331767 10.1111/j.1365-294X.2009.04480.x

[ece374283-bib-0046] Taberlet, P. , E. Coissac , F. Pompanon , C. Brochmann , and E. Willerslev . 2012. “Towards Next‐Generation Biodiversity Assessment Using DNA Metabarcoding.” Molecular Ecology 21, no. 8: 2045–2050. 22486824 10.1111/j.1365-294X.2012.05470.x

[ece374283-bib-0047] Thomsen, P. F. , and E. Willerslev . 2015. “Environmental DNA–an Emerging Tool in Conservation for Monitoring Past and Present Biodiversity.” Biological Conservation 183: 4–18.

[ece374283-bib-0048] Tsipoura, N. , and J. Burger . 1999. “Shorebird Diet During Spring Migration Stopover on Delaware Bay.” Condor 101, no. 3: 635–644.

[ece374283-bib-0049] Tsuji, S. , T. Takahara , H. Doi , N. Shibata , and H. Yamanaka . 2019. “The Detection of Aquatic Macroorganisms Using Environmental DNA Analysis—A Review of Methods for Collection, Extraction, and Detection.” Environmental DNA 1, no. 2: 99–108.

[ece374283-bib-0051] U.S. Fish and Wildlife Service . 2021. “Endangered and Threatened Wildlife and Plants; Proposed Revised Critical Habitat for the Rufa Red Knot ( *Calidris canutus rufa* ).” Federal Register 86, no. 133: 37526–37589. https://www.federalregister.gov/documents/2021/07/15/2021‐14406/endangered‐and‐threatened‐wildlife‐and‐plants‐proposed‐revised‐critical‐habitat‐for‐the‐rufa‐red‐knot.

[ece374283-bib-0052] Van Gils, J. A. , T. Piersma , A. Dekinga , and M. W. Dietz . 2003. “Cost‐Benefit Analysis of Mollusk‐Eating in a Shorebird II. Optimizing Gizzard Size in the Face of Seasonal Demands.” Journal of Experimental Biology 206, no. 19: 3369–3380. 12939369 10.1242/jeb.00546

[ece374283-bib-0053] Vestheim, H. , and S. N. Jarman . 2008. “Blocking Primers to Enhance PCR Amplification of Rare Sequences in Mixed Samples – A Case Study on Prey DNA in Antarctic Krill Stomachs.” Frontiers in Zoology 5, no. 12: 12. 10.1186/1742-9994-5-12. 18638418 PMC2517594

[ece374283-bib-0056] Zwarts, L. , and J. H. Wanink . 1993. “How the Food Supply Harvestable by Waders in the Wadden Sea Depends on the Variation in Energy Density, Body Weight, Biomass, Burying Depth, and Behavior of Tidal‐Flat Invertebrates.” Netherlands Journal of Sea Research 31, no. 4: 441–476.

