# Supplementary material for: Developing a Fecal DNA Method With Blocking Primers to Determine Red Knots' (Calidris canutus) Diets Noninvasively
Source: Ecol Evol. 2026 Sep 15;16(9):e74283. doi: 10.1002/ece3.74283 (PMC13578112; doi:10.1002/ece3.74283)
Supplement: Supplementary file 1 [file ECE3-16-e74283-s001.docx]

**Supplementary Data**

**Supplementary Table 1**. Summary of Red Knot and other animal taxa across samples in the Blocker Test.

| **Sample** | **Blocker** | **% Red Knot Reads** | **# Red Knot Reads** | **# Other Animal Reads** | **# Other Animal Taxa** |
| --- | --- | --- | --- | --- | --- |
| **M1-feces** | no | 99.3% | 29,474 | 201 | 6 |
| **M1-feces** | yes | 78.7% | 2,592 | 702 | 11 |
| **M2-feces** | no | 33.3% | 4,452 | 8,914 | 25 |
| **M2-feces** | yes | 0.1% | 13 | 10,545 | 21 |
| **A1-feces** | no | 97.9% | 52,901 | 1,132 | 13 |
| **A1-feces** | yes | 65.1% | 8,602 | 4,617 | 15 |
| **A2-feces** | no | 96.9% | 15,603 | 507 | 9 |
| **A2-feces** | yes | 14.6% | 174 | 1,018 | 8 |
| **M3-sediment** | no | 0.0% | 4 | 20,436 | 7 |
| **M3-sediment** | yes | 0.0% | 0 | 25,940 | 9 |
| **M4-sediment** | no | 0.0% | 1 | 22,848 | 18 |
| **M4-sediment** | yes | 0.0% | 0 | 16,700 | 19 |
| **A3-sediment** | no | 0.0% | 0 | 2,056 | 13 |
| **A3-sediment** | yes | 0.0% | 0 | 3,288 | 16 |
| **A4-sediment** | no | 0.0% | 3 | 26,847 | 21 |
| **A4-sediment** | yes | 0.0% | 0 | 22,849 | 17 |

**Supplementary Figure 1:** Rarefaction (ASV accumulation) curves for the extraction-kit test samples. This was generated in R version 4.3.2.
